# *Escherichia coli* Isolated from Urinary Tract Infections of Lebanese Patients between 2000 and 2009: Epidemiology and Profiles of Resistance

**DOI:** 10.1155/2011/218431

**Published:** 2011-10-12

**Authors:** Ziad Daoud, Claude Afif

**Affiliations:** ^1^Faculty of Medicine and Medical Sciences, University of Balamand, P.O. Box 100, Tripoli, Lebanon; ^2^Faculty of Medicine and Medical Sciences, University of Balamand, P.O. Box 166378, Beirut, Lebanon

## Abstract

The purpose of this study was to investigate the bacterial etiology of urinary tract infections in one of the busiest hospitals of Lebanon and to examine the epidemiologic and microbiologic properties of *Escherichia coli* isolated from urinary tract infections of Lebanese patients over a 10-year period. *Methods*. This retrospective study analyzed the data generated between 2000 and 2009 (10,013 Gram-positive and Gram-negative bacteria). Bacterial identification was based on standard culture and biochemical characteristics of isolates. Antimicrobial susceptibility was tested by the disk diffusion method, and ESBL production was detected by synergy with third-generation cephalosporins and amoxiclav. *Results*. *E. coli* was the most frequent isolate throughout the ten years (60.64% of the total isolates). It was followed by *Klebsiella pneumoniae* and *Proteus sp., Pseudomonas aeruginosa, Enterococcus sp.*, and *Streptococcus agalactiae*. *E. coli* occurred more frequently in women (69.8%) than in men (61.4%). The lowest percentage of susceptibility of *E. coli* was manifested against piperacillin and ampicillin. An increase in the production of ESBL was observed (2.3% in 2000 to 16.8% in 2009). *Conclusions*. The etiology of urinary tract infections and their susceptibility profiles are important to be evaluated in countries like Lebanon where a severe misuse of antibiotics at all levels is observed.

## 1. Introduction

Urinary tract infections (UTIs) are one of the most common infectious diseases [1–3]. They may be symptomatic or asymptomatic, and either type of infection can result in serious sequelae if not appropriately treated [4]. Although different causative agents can be responsible for UTIs, bacteria are the major cause being responsible for more than 95% of UTI cases [5]. In this context, *E. coli* is the most prevalent organism and is solely responsible for the majority of these infections. An accurate and prompt diagnosis is important in shortening the disease course and for preventing the ascent of the infection to the upper urinary tract [6]. Treatment of UTI is often started empirically. UTIs are often treated with different broad-spectrum antibiotics when one with a narrow spectrum of activity may be appropriate because of concerns about infection with resistant organisms, and antimicrobial susceptibility testing of the urinary pathogens constitutes the basis for antibiotic therapy. However, in view of the increasing bacterial resistance, regular monitoring of resistance patterns is necessary to improve guidelines for empirical antibiotic therapy [6–8]. The present study aims at analyzing the infectious epidemiology of UTIs in a general university hospital located in Beirut over a period of ten years. In addition, it examines the susceptibility profiles of *E. coli* between 2000 and 2009.

## 2. Material and Methods

### 2.1. Study Design

This is a retrospective study conducted at the Microbiology section of the Medical Laboratories of the Saint George Hospital-University Medical Center in Beirut and covering ten years (2000 to 2009). The population included all in- and outpatients with documented UTI. This included 10,013 different Gram-positive and Gram-negative bacteria in addition to *Candida albicans* and *Candida sp.* There were 6,708 (66.99%) samples from female patients and 3,305 (33.01%) from male patients. Adult patients were sampled by clean catch midstream urine, and children aged less than 3 years were sampled using sterile urine bags. Only a single positive culture per patient was included in the analysis within the period of three months. 

### 2.2. Isolation and Identification of Organisms

Samples for urine culture were tested within half an hour of sampling. All samples were inoculated on blood agar as well as Mac Conckey agar and incubated at 37°C for 24 hours, and for 48 hours in negative cases. A specimen was considered positive for UTI in the light of the number of yielded colonies (≥10^5^ cfu/mL) and the cytology of the urine through microscopic detection of bacteriuria and PMNs (≥8 leukocytes/mm^3^). However, lower colony counts associated with significant pyuria or low PMN count associated with significant colony counts was considered and analyzed in the light of the clinical picture and the patient's immunological status. Bacterial identification was based on standard culture and biochemical characteristics of isolates.

Gram-negative bacteria were identified by standard biochemical tests [5, 6]. Gram-positive microorganisms were identified with the corresponding recommended laboratory tests.

### 2.3. Susceptibility Testing

Antimicrobial susceptibility of *E. coli* was tested by the disk diffusion method according to the CLSI recommendations, using the Mueller-Hinton agar [6]. Antimicrobial agents tested were ampicillin, amoxicillin-clavulanic acid, aztreonam, cephalothin, cefoxitin, cefuroxime, cefotaxime, ceftriaxone, ceftazidime, cefipime, piperacillin, piperacillin-tazobactam, imipenem, gentamycin, tobramycin, norfloxacin, ciprofloxacin, trimethoprim-sulfamethoxazole, and tetracycline. The CLSI-ESBL phenotypic confirmatory test with ceftazidime, cefotaxime, ceftriaxone, and cefixime was performed for all the isolates by disk diffusion method on the Mueller-Hinton agar plates with and without 10 *μ*g of amoxiclav. Susceptibility test results were interpreted according to the criteria established by the Clinical & Laboratory Standard Institute (CLSI). A minimum of 5 mm increase in the zone of diameter of third-generation cephalosporins, tested in combination with amoxiclav versus its zone when tested alone, was considered indicative of ESBL production. *E. coli *ATCC 25922 was used as ESBL-negative and *K. pneumoniae *700603 was used as ESBL-positive reference strain. Statistical analysis: variables were expressed as percentages.

## 3. Results

Over a 10-year period, a total of 10,013 positive urine isolates including 6,071 *E. coli* were analyzed. 

Tables 1 and 2 show the microorganisms most frequently isolated from these positive urine cultures. As expected, *E. coli* was the most frequent isolate throughout the ten years (average of 60.64% of the total isolates). It was followed by *Klebsiella pneumoniae* where rate of isolation ranged between 6.1 and 9.9%. The next most frequently isolated bacteria were *Proteus sp., Pseudomonas aeruginosa, Enterococcus sp., *and* Streptococcus agalactiae*. *Candida albicans* and *Candida sp.* were commonly isolated; however, their clinical significance was not always evident. If *Candida* and minor bacterial isolates are not included, Gram-negative bacteria accounted for 92% of the UTI, while Gram-positive infections were responsible only for 8%. 

Analysis of the results according to patient gender (not shown) indicated that although *E. coli* is the predominant isolated pathogen from both sexes, it occurred more frequently in women (69.8% in women compared to 61.4% in men.) The trend of infectious etiology in the population did not really differ over the 10-year period.

The percentages of susceptibility (and subsequently of nonsusceptibility including both resistant and intermediately resistant strains) of *E. coli* isolates to the panel of antibiotics which are commonly used to treat *Escherichia *infections are shown in Table 3. The lowest percentage of susceptibility was manifested against piperacillin (between 9 and 24%) followed by ampicillin (between 26 and 38%), whereas an absolute susceptibility was observed with imipenem (100%). In general, the urinary isolates showed a slightly better susceptibility profile in comparison to all the hospital isolates of *E. coli*. Over the successive years, the susceptibility to cephalosporins, including generations 3 and 4, tends to decrease; this is coupled by an increase in the production of ESBL as shown in Table 4 where ESBL production goes from 2.3% in 2000 to 16.8% in 2009 for the urinary isolates.

Table 5 shows the susceptibility profiles of ESBL producing *E. coli* to families of antibiotics other than *beta*-lactams. Tigecycline, amikacin, and piperacillin-tazobactam seem to have the highest antibacterial activity on these organisms.

Table 6 shows the percentages of simultaneous resistance in the urinary isolates of *E. coli *to various families of antimicrobial agents.

## 4. Discussion

This study shows the distribution of microbial species and antibiotic susceptibility patterns of *E. coli* isolated from Lebanese patients with UTIs. Saint George Hospital is a 300-bed hospital located in Beirut; however, it is one of the busiest hospitals in the country and receives patients from different areas of Lebanon. In this retrospective study, no clinical data are provided, and this constitutes by itself a limitation. Subsequently, important information related to symptomatic versus asymptomatic, complicated versus uncomplicated UTI, and health-care-associated versus catheter-related UTIs could not be addressed and discussed. The majority of pathogens were isolated from women (69.8%). It has been extensively reported that adult women have a higher prevalence of UTI than men, principally owing to anatomic and physical factors [9].

Antibiotic resistance is a major clinical problem in treating infections caused by these microorganisms. The resistance to the antimicrobials has increased over the years. Resistance rates vary from country to country [10]. In Lebanon, there is an evidence for increase in ESBL producing Enterobacteriaceae. This was previously reported in our hospital as well as in other institutions in the country [11–15]

In this study, *E. coli* accounted for approximately 61% of all clinically significant urinary isolates and 76.8% of all Enterobacteriaceae. This is consistent with the findings of previous studies in which *E. coli *was the predominant pathogen isolated from patients with UTIs [16, 17]. The rate of isolation of *Klebsiella pneumoniae *might be described as high when compared to other studies [16, 17]; however, this can be explained by the fact that our study includes both hospital and acquired UTI. 

*E. coli *isolates from urinary infections show a similar pattern of susceptibility to those isolated from all body site infections although with a more enhanced susceptibility percentages. Aminopenicillins do not constitute a therapeutic option in this population; even the combination amoxicillin-clavulanic acid does not seem to offer important alternative for treatment. In view of the increasing ESBL production, all cephalosporins activities are affected and challenged by these inactivating enzymes. Imipenem remains the only antibiotic with 100% of susceptibility. 

It is pretty alarming to note that between 7.3 and 13.8% of the urinary isolates of *E. coli *show simultaneous resistance to third-generation cephalosporins, aminoglycosides, and fluoroquinolones. A genetic investigation on these isolates should be performed in order to identify the mechanisms of resistance and discover whether they are correlated to each other or independently occurring.

## Figures and Tables

**Table 1:** The organisms most commonly isolated from Lebanese patients with UTI between 2000 and 2004 at Saint George Hospital University Medical Center, Beirut.

	The microorganisms most commonly isolated from Lebanese patients with UTI between 2000 and 2004									
	2000		2001		2002		2003		2004	
	Nb	%	Nb	%	Nb	%	Nb	%	Nb	%
*Acinetobacter sp.*	3	0.5	4	0.5	2	0.2	0	0.0	14	1.3
*Candida albicans*	15	2.4	12	1.6	23	2.4	26	2.4	31	2.9
*Candida sp.*	16	2.5	22	2.9	28	2.9	47	4.3	34	3.1
*Citrobacter sp.*	6	0.9	11	1.5	19	2.0	17	1.5	15	1.4
*Enterobacter sp.*	4	0.6	12	1.6	14	1.5	6	0.5	16	1.5
*Enterococcus faecalis*	17	2.7	18	2.4	28	2.9	48	4.4	20	1.9
*Enterococcus faecium*	6	0.9	18	2.4	20	2.1	30	2.7	35	3.2
*Escherichia coli*	395	62.5	440	58.3	575	59.8	637	57.9	661	61.1
*Klebsiella sp.*	48	7.6	75	9.9	59	6.1	85	7.7	79	7.3
*Morganella morganii*	5	0.8	4	0.5	11	1.1	14	1.3	14	1.3
*Proteus sp.*	45	7.1	50	6.6	72	7.5	74	6.7	65	6.0
*Pseudomonas aeruginosa*	30	4.7	38	5.0	45	4.7	35	3.2	51	4.7
*Pseudomonas sp.*	0	0.0	2	0.3	1	0.1	1	0.1	0	0.0
*Staphylococcus aureus*	3	0.5	6	0.8	3	0.3	7	0.6	9	0.8
*Staphylococcus saprophyticus*	6	0.9	7	0.9	5	0.5	2	0.2	3	0.3
*Streptococcus agalactiae*	20	3.2	15	2.0	14	1.5	25	2.3	18	1.7
*Streptococcus, Group D*	1	0.2	2	0.3	4	0.4	1	0.1	6	0.6
Other minor organisms	12	1.9	19	2.5	38	4.0	45	4.1	10	0.9

Total isolates	632		755		961		1100		1081	

**Table 2:** The organisms most commonly isolated from Lebanese patients with UTI between 2000 and 2004 at Saint George Hospital University Medical Center, Beirut.

	The microorganisms most commonly isolated from Lebanese patients with UTI between 2005 and 2009									
	2005		2006		2007		2008		2009	
	Nb	%	Nb	%	Nb	%	Nb	%	Nb	%
*Acinetobacter sp.*	19	1.9	14	1.2	18	1.6	16	1.3	5	0.5
*Candida albicans*	14	1.4	37	3.3	27	2.4	29	2.4	25	2.5
*Candida sp.*	29	2.9	57	5.1	34	3.0	31	2.6	35	3.5
*Citrobacter sp.*	15	1.5	18	1.6	19	1.7	16	1.3	9	0.9
*Enterobacter sp.*	16	1.6	22	2.0	14	1.2	19	1.6	6	0.6
*Enterococcus faecalis*	14	1.4	15	1.3	20	1.8	14	1.2	15	1.5
*Enterococcus faecium*	32	3.2	25	2.2	34	3.0	34	2.8	6	0.6
*Escherichia coli*	609	60.2	711	63.4	688	60.7	727	60.0	628	62.5
*Klebsiella sp.*	92	9.1	84	7.5	100	8.8	118	9.7	90	9.0
*Morganella morganii*	13	1.3	6	0.5	10	0.9	24	2.0	7	0.7
*Proteus sp.*	57	5.6	53	4.7	70	6.2	73	6.0	63	6.3
*Pseudomonas aeruginosa*	39	3.9	27	2.4	40	3.5	44	3.6	41	4.1
*Pseudomonas sp.*	0	0.0	6	0.5	0	0.0	6	0.5	3	0.3
*Staphylococcus aureus*	7	0.7	4	0.4	6	0.5	4	0.3	10	1.0
*Staphylococcus saprophyticus*	0	0.0	5	0.4	1	0.1	2	0.2	3	0.3
*Streptococcus agalactiae*	27	2.7	14	1.2	16	1.4	16	1.3	8	0.8
*Streptococcus, Group D*	1	0.1	1	0.1	1	0.1	0	0.0	1	0.1
Other minor organisms	28	2.8	23	2.0	36	3.2	38	3.1	50	5.0

Total isolates	1012		1122		1134		1211		1005	

**Table 3:** Susceptibility profiles of urinary and nonurinary isolates of *E. coli* collected from Lebanese patients between 2000 and 2009 at Saint George Hospital University Medical Center, Beirut.

			Percentages of susceptibility of *E. coli* isolated from Lebanese patients																	
Year	Site of isolation	Number	Antibacterial Agent																	
			Ampicillin	Amox-Clav	Aztreonam	Cephalothin	Cefoxitin	Cefuroxime	Cefotaxim	Ceftriaxone	Ceftazidime	Cefepime	Piperacillin	Pipera-Tazo	Imipenem	Gentamycin	Tobramycin	Norfloxacin	Ciprofloxacin	Trimeth-Sulfa
2000	General	620	37	58	95	54	92	89	96	96	95	97	20	93	100	89	88	75	78	51
	Urinary	395	38	59	98	57	97	95	96	96	97	99	24	94	100	92	89	88	83	54
	Nonurinary	225	35	56	91	52	81	82	95	95	94	96	17	93	100	85	88	59	70	52
2001	General	697	28	56	92	49	91	82	93	93	92	95	18	93	100	89	88	75	78	48
	Urinary	440	32	63	94	51	92	88	96	96	97	97	22	94	100	92	89	88	83	51
	Nonurinary	257	22	49	91	47	89	74	91	89	87	92	13	91	100	87	86	61	73	45
2002	General	901	30	62	92	60	96	84	94	94	92	96	12	96	100	83	79	64	76	50
	Urinary	699	34	69	94	62	97	90	96	97	97	97	16	97	100	86	80	77	81	53
	Nonurinary	202	26	54	89	56	93	77	90	91	87	94	7	93	100	81	77	50	73	45
2003	General	1221	28	58	81	54	96	83	83	83	82	83	13	95	100	78	74	60	77	48
	Urinary	637	32	65	83	56	97	89	89	90	89	90	17	96	100	81	75	73	82	51
	Nonurinary	516	24	50	78	51	94	77	76	80	74	77	10	94	100	75	74	48	72	44
2004	General	1094	28	65	52	59	95	77	82	82	82	82	9	99	100	78	81	62	65	64
	Urinary	661	32	72	54	61	96	83	88	89	89	89	13	100	100	81	82	75	70	67
	Nonurinary	433	25	59	50	56	93	71	75	75	75	74	7	97	100	78	79	52	63	60
2005	General	998	26	61	53	49	95	76	80	80	80	81	10	98	100	76	99	58	62	51
	Urinary	609	30	68	55	51	96	82	86	87	87	88	14	99	100	79	100	71	67	54
	Nonurinary	389	24	55	51	48	93	72	75	73	72	74	8	97	100	74	99	51	60	49
2006	General	1072	29	64	79	54	95	80	83	83	83	83	10	99	100	79	99	43	71	49
	Urinary	711	33	71	81	56	96	86	89	90	90	90	14	100	100	82	100	56	76	52
	Nonurinary	361	25	60	79	55	94	75	79	78	78	78	8	99	100	76	99	39	74	48
2007	General	1049	29	65	81	59	95	78	81	81	81	81	10	99	100	80	99	44	48	47
	Urinary	688	34	72	83	61	96	84	87	88	88	88	14	100	100	83	100	57	53	50
	Nonurinary	361	23	60	80	58	93	73	78	77	77	77	7	99	100	78	99	36	45	44
2008	General	1098	28	66	77	55	89	73	78	80	80	80	9	99	100	75	93	42	47	45
	Urinary	727	30	67	80	55	96	78	82	88	88	89	15	99	100	82	98	62	52	51
	Nonurinary	371	25	65	76	54	84	69	75	73	73	73	5	98	100	70	89	39	45	40
2009	General	1011	26	60	79	46	96	76	79	79	79	79	13	98	100	79	99	44	46	47
	Urinary	628	31	69	81	47	96	84	86	86	87	53	18	99	100	83	99	63	52	51
	Nonurinary	383	23	53	79	45	96	69	74	74	75	80	11	99	100	76	99	35	41	44

**Table 4:** ESBL production in *E. coli* collected from Lebanese patients between 2000 and 2009 at the Saint George Hospital University Medical Center, Beirut. U: urinary isolates, NU: nonurinary isolates.

	ESBL production in *E. coli* isolated between 2000 and 2009														
	2000			2001			2002			2003			2004		
Isolates	All	U	NU	All	U	NU	All	U	NU	All	U	NU	All	U	NU
Number of isolates	620	395	225	697	440	257	901	699	202	1221	637	584	1094	661	433
% of ESBL	2.3	2.1	2.3	4	3.9	4.3	9.8	9.9	9.5	13.6	14.5	12.1	12.9	11.0	14.1
	2005			2006			2007			2008			2009		

Isolates	All	U	NU	All	U	NU	All	U	NU	All	U	NU	All	U	NU

Number of isolates	998	609	389	1072	711	361	1049	688	361	1098	727	371	1011	628	383
% of ESBL	20.3	15.7	26.1	17.4	15.6	19.1	19.45	19.2	19.8	19.4	17.1	22.0	18.6	16.8	21.8

**Table 5:** Susceptibility profiles of ESBL producing *E. coli* collected from Lebanese patients between 2005 and 2009 at the Saint George Hospital-University Medical Center, Beirut.

		Percentages of susceptibility of ESBL producing *E. coli *						
		Antibacterial agent						
Year	Isolates	Amikacin	Cefoxitin	Gentamycin	Pip + Tazo	TSM	Tigecycline	Ciprofloxacin
2005	All	90.5	85.6	29.8	97.6	39.4	ND	21.1
	Urinary	92.3	87.6	35.2	97.5	41.1	ND	16.4
	Nonurinary	88.2	83.5	25.1	97.8	37.1	ND	17.9
2006	All	97.9	80.7	27.3	93.3	24.1	ND	17.6
	Urinary	95.6	84.4	33.5	94.1	40.8	ND	18.8
	Nonurinary	98.3	76.9	22.1	93.5	18.9	ND	16.5
2007	All	93.6	81.4	30.1	92.2	23.5	89.6	23.1
	Urinary	91.2	87.5	22.4	89.9	36.8	91.2	25.7
	Nonurinary	90.2	75.6	36.8	95.4	41.3	93.2	19.1
2008	All	94.2	80.6	27.8	94.1	25.6	87.4	18.8
	Urinary	89.1	82.4	24.4	87.9	26.6	91.4	21.1
	Nonurinary	97.3	77.1	32.2	98.8	23.9	82.1	15.5
2009	All	93.6	83.4	30.8	94.2	19.5	91.6	19.6
	Urinary	94.6	84.5	36.1	96.6	25.4	94.4	19.5
	Nonurinary	91.8	82.2	26.3	92.2	10.6	89.9	19.2

**Table 6:** Multiple resistance of urinary isolates of *E. coli. *

	Percentage of resistance profiles				
Resistance profile of urinary *E. coli *	2005	2006	2007	2008	2009
Resistant to aminoglycosides and fluoroquinolones	3.2	2.6	3.1	3	3.7
Resistant to 3rd-Generation cephalosporins and fluoroquinolones	3.6	3	4.9	8.2	9.6
Resistant to 3rd-Generation cephalosporins and aminoglycosides	3.9	3.4	3.5	2.5	3.1
Resistant to 3rd-Generation cephalosporins, aminoglycosides, and fluoroquinolones	8.7	9	9.8	13.8	7.3
